# Safety and Effectiveness of Multi-Switch Between Adalimumab Originator and Biosimilars: A Multicenter (SUSTAIN) Study

**DOI:** 10.3390/jcm14248819

**Published:** 2025-12-12

**Authors:** Mohammad Shehab, Anwar Almajdi, Israa Abdullah, Fatema Alrashed

**Affiliations:** 1 Program of Medicine, College of Medicine and Health Sciences, Abdullah Al Salem University, Khaldiya 42167, Kuwait; 2 Division of Gastroenterology and Hepatology, McGill University Health Center, Montreal, QC H3G 2M1, Canada; 3 Division of Gastroenterology, Department of Internal Medicine, Mubarak Alkabeer, University Hospital, Jabriya 47060, Kuwait; almajdi.anwar@gmail.com (A.A.);; 4 Department of Pharmacy Practice, College of Pharmacy, Kuwait University, Jabriya 13110, Kuwait; fatema.alrashed@ku.edu.kw

**Keywords:** ulcerative colitis, Crohn’s disease, biosimilar, adalimumab

## Abstract

**Background/Objectives**: Biologic therapies have transformed the management of inflammatory bowel disease (IBD), but their high cost has prompted the introduction of biosimilars. Although switching from biologic originators to biosimilars is increasingly common, real-world evidence remains limited. We aimed to explore the safety and efficacy of switching between biologic originators and biosimilars. **Methods**: We conducted a retrospective chart review of patients with IBD between 2015 and 2025. Adult patients receiving adalimumab-adaz or adalimumab-atto were included. Patients who were non-medically switched once from adalimumab originator (Humira^®^) to any biosimilar were classified as group A. Patients who also switched back to originator (multiple switches) were classified as group B. The outcomes of the study were safety and efficacy of the biosimilars. Logistic regression identified switching predictors. **Results**: A total of 237 patients were included in the study. The number of patients in group A and group B was 208 and 58 patients, respectively. Sustained clinical remission was achieved in 198 (95.4%) of group A and 54 (93.6%) of group B participants. Sustained normalization of inflammatory markers was also comparable, occurring in 190 (91.5%) of group A and 54 (92.3%) of group B participants. No treatment-emergent AEs, infections, or treatment discontinuations were reported in either group (0%). Regression analysis identified older age and prior immunomodulator use as significant predictors of switching. **Conclusions**: Multiple switches of adalimumab biosimilars can be safely undertaken without increasing the risk of adverse reactions or treatment failure. This study provides meaningful evidence to guide policy and physician confidence in biosimilar interchangeability as a sustainable IBD therapeutic strategy.

## 1. Introduction

Inflammatory bowel disease (IBD), which includes Crohn’s disease (CD) and ulcerative colitis (UC), is a chronic inflammatory condition [1]. The management of IBD has undergone a significant transformation with the advent of biologic therapies, which have proven to be highly effective in controlling disease activity, inducing remission, and improving quality of life for patients. These biologics, primarily tumor necrosis factor (TNF) inhibitors, integrin inhibitors, and interleukin inhibitors, have revolutionized the treatment landscape by targeting specific molecules involved in the inflammatory process [2,3].

However, the high cost of biologic medications has driven the development and use of biosimilars, which are biologically similar drugs that are developed to match an approved reference biologic in terms of structure, efficacy, and safety [4]. Biosimilars offer a more affordable alternative while maintaining therapeutic efficacy, presenting an opportunity to enhance access to biologic therapies for a broader patient population.

In recent years, there has been increasing interest in the practice of switching from an originator biologic to a biosimilar, a strategy that has been particularly relevant in the treatment of IBD [5]. While switching offers economic benefits and broadens patient access to treatment, it also raises questions about clinical outcomes, immunogenicity, safety, and patient preference. The European Crohn’s and Colitis Organization (ECCO) has stated that prescribing biosimilars and switching from originators to biosimilars in patients with IBD are acceptable, provided that patients are well-informed and adequately monitored [6]. This transition involves careful consideration of various factors including the disease’s activity, the patient’s treatment history, and the scientific evidence surrounding the safety and efficacy of biosimilars in IBD management.

While real-world data suggest that both single and double switches with infliximab maintain effectiveness and safety [4], comparable evidence for adalimumab remains limited. In the current economic landscape, where multiple biosimilars are available at competitive costs, robust data on the outcomes of multiple biosimilar switches has become increasingly relevant. Furthermore, while the safety and efficacy of adalimumab biosimilars are well-established globally, real-world data from the Middle East and Gulf region is still limited. Regional differences in genetic background, environmental exposures, healthcare delivery, and paucity of local data in the IBD population necessitate local validation to ensure confidence in treatment outcomes [7,8]. Therefore, this study aims to explore the safety and efficacy of single and multiple switching between adalimumab originator and adalimumab biosimilars in the treatment of IBD. Additionally, we aimed to investigate the predictors of switching between adalimumab originator and biosimilar therapies.

## 2. Materials and Methods

### 2.1. Patient Population

This is a retrospective study that was conducted in two specialized gastroenterology tertiary centers: Mubarak Al-Kabeer hospital and Alfarwaniya hospital. It was reported in accordance with Strengthening the Reporting of Observational Studies in Epidemiology (STROBE) guidelines [9].

The patient enrollment period was between September 2020 and September 2025. Inclusion criteria were as follows: adult patients aged 18 years or older, diagnosed with moderate to severe Crohn’s disease (CD) or ulcerative colitis (UC), who have been on adalimumab originator (Humira^®^) for at least 12 months before enrolment. Patients should be in clinical remission with normal inflammatory markers (C-reactive protein (CRP) < 10 mg/L, fecal calprotectin < 250 µg/g, or albumin > 35 g/L) before switching to one of the available biosimilar agents: adalimumab-adaz or adalimumab-atto. Clinical remission in CD was defined as a Crohn’s disease Activity Index (CDAI) score <150 or Harvey–Bradshaw Index (HBI) < 5 [10,11], whereas in UC, it was defined as a partial Mayo score ≤ 2 [12].

Exclusion criteria included pregnant females, patients with concomitant autoimmune diseases (such as psoriasis), and patients who received other biologic or small-molecule therapy for any autoimmune conditions, e.g., rheumatological disease. Additionally, patients who were receiving or had received immunomodulators or corticosteroids < 12 weeks before enrolment were excluded. Finally, patients with missing data and those without follow-up data were excluded.

Diagnosis was made according to the international classification of diseases (ICD-11 version 2024). Patients were considered to have IBD when they had ICD-11 DD70 corresponding to CD and ICD-DD71 corresponding to UC [13].

Ethical approval for this study was obtained on 11 July 2024 from the standing committee for the coordination of health and medical research at the ministry of health of Kuwait (IRB No. 105 5/2024) and as per the updated guidelines of the Declaration of Helsinki (64th WMA General Assembly, Fortaleza, Brazil, October 2013) and the US Federal Policy for the Protection of Human Subjects.

This study collected baseline and clinical data from patients with IBD, including demographic information such as age, sex, and the biosimilar name of the biologic agent used for treatment. The type of IBD was classified as either UC or CD, with disease localization specified according to the Montreal classification as L1: disease is limited to the terminal ileum, L2: disease is limited to the colon, L3: disease is in the ileum and colon (ileocolonic), and L4: upper gastrointestinal (UG) involvement for CD or E1: proctitis, E2: left-sided colitis, and E3: extensive/pancolitis for UC. In the case of CD, disease behavior was further categorized as B1: non-stricturing, non-penetrating disease, B2: stricturing disease, B3: penetrating disease, and P: perianal disease. Additional clinical characteristics, including comorbidities (yes/no), were also recorded, along with the duration of diagnosis in years. Treatment history was assessed with a particular focus on biologic therapy, including whether the patient had previously used biologics, the number of biologics previously prescribed, and the total duration of biologic therapy (in months). The use of immunomodulators or corticosteroids 3 to 12 months prior to biosimilar initiation were documented. Finally, additional laboratory data collected were levels of CRP, fecal calprotectin, and albumin.

Patients were divided into group A and group B. Patients who were non-medically switched from adalimumab originator (Humira^®^) to adalimumab biosimilar and maintained on it for at least 6 months were classified as group A. Patients who were non-medically switched from adalimumab originator (Humira^®^) to adalimumab biosimilar and then non-medically switched back to the adalimumab originator within 6 months from the first switch were classified as group B (multiple switches). Group B must have remained on the originator after switching back for at least 6 months.

### 2.2. Primary Outcome

The primary outcome of this study was to assess the safety of a single switch (group A) and multiple switching (group B) to and from biosimilar agents over a 6-month period. This duration is considered sufficient to capture the majority of adverse events and safety outcomes.

The frequency, severity, and type of adverse events (AEs) were reported. This includes treatment-emergent AEs defined as events with onset after the first dose of biosimilar and within the study period. These can include any serious AEs, such as infections, or severe AEs leading to the discontinuation of the biosimilar. Local/mild AEs such as injection site reactions were also reported. Finally, all AEs were reported, regardless of a possible relationship to the study drug.

### 2.3. Secondary Outcome

Secondary outcomes were assessing efficacy measures of a single switch (group A) and multiple switching (group B) to and from biosimilar agents over 6 months. The efficacy end points assessed were the proportion of CD patients who continued to be in clinical remission (sustained clinical remission), as indicated by an HBI < 5 or a CDAI score < 150, and the proportion of UC patients who achieved remission, as indicated by a partial Mayo score of ≤2. An additional secondary outcome was the proportion of patients with sustained normalization of inflammatory markers; CRP < 10 mg/L, fecal calprotectin < 250 µg/g, or albumin > 35 g/L).

### 2.4. Statistical Analysis

Statistical analyses were performed using IBM SPSS Statistics (Version 29.0, IBM Corp., Armonk, NY, USA). Baseline demographic and clinical characteristics were summarized using appropriate descriptive statistics. Continuous variables were expressed as means with standard deviations or medians with interquartile ranges (IQRs), depending on data distribution. Categorical variables were reported as frequencies and percentages. A chi-square test was conducted to compare outcomes between group A and group B, assessing whether there were statistically significant differences in the proportions of patients achieving clinical remission and normalization of inflammatory markers and experiencing adverse events. Logistic regression analysis was conducted to identify potential associations between biosimilar switching and patient characteristics, including age, sex, IBD type, and prior immunomodulator or corticosteroid administration within 3 to 12 months before biosimilar initiation. Results were expressed as Odds Ratios (ORs) with 95% confidence intervals (CIs). Statistical significance was established at *p* < 0.05.

## 3. Results

### 3.1. Cohort Characteristics

**Figure 1:**
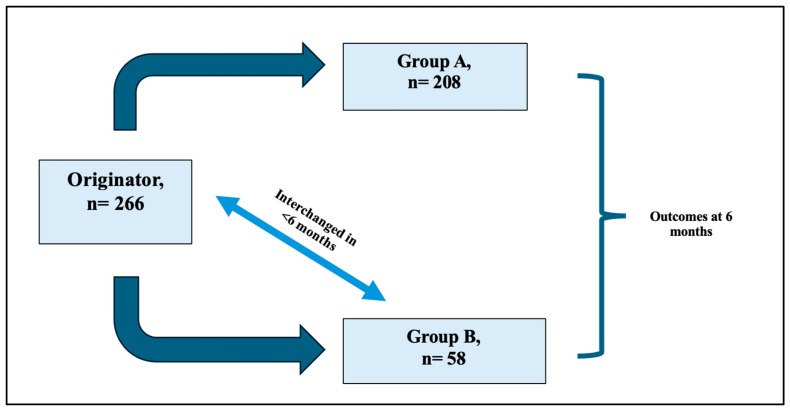
Diagram showing cohort distribution between group A and group B (multiple switches).

More than 500 patients’ medical profiles were screened. A total of 237 patients with IBD were included in the study. The number of patients who were non-medically switched from adalimumab originator (Humira^®^) to adalimumab biosimilar (group A) was 208 patients, of whom 187 (90%) had CD and 21 (10%) had UC. The number of patients who were non-medically switched from adalimumab originator (Humira^®^) to adalimumab biosimilar and non-medically switched back to the adalimumab originator (group B) was 58 patients, of whom 53 (92%) had CD and 5 (8%) had UC (Figure 1).

The median age of the participants was 32 years (IQR 26–43.50) with 61.6% (n = 146) of the patients being male. The majority of patients in the cohort had Crohn’s disease (CD) (n = 209, 88.19%), while 28 patients (11.81%) had ulcerative colitis; the median disease duration was 7 years (IQR, 3–12). The majority of this study population switched from adalimumab originator to adalimumab-adaz [204 (86.1%)], while the rest were switched to the biosimilar adalimumab-atto [33 (3.9%)]. Cohort characteristics are described in Table 1.

Most patients (n = 224, 94.5%) had received biologic therapy before, with a median duration of biologic use of 65 months (IQR, 28–112). Nearly half of the patients were on immunomodulators before starting the biosimilar therapy (n = 110, 46.4) and a total of 26 patients (10.97%) had received corticosteroids within the 12 months preceding the biosimilar initiation.

### 3.2. Outcome Measures

No treatment-emergent adverse events (AEs), infections, or treatment discontinuations were reported in either group (0%). Local or mild AEs were rare, occurring in three (1.2%) participants in group A and one (2%) in group B, with no other AEs reported (0% in both groups). Overall, both groups demonstrated comparable efficacy and tolerability profiles, with minimal adverse effects (Table 2).

**Figure 2:**
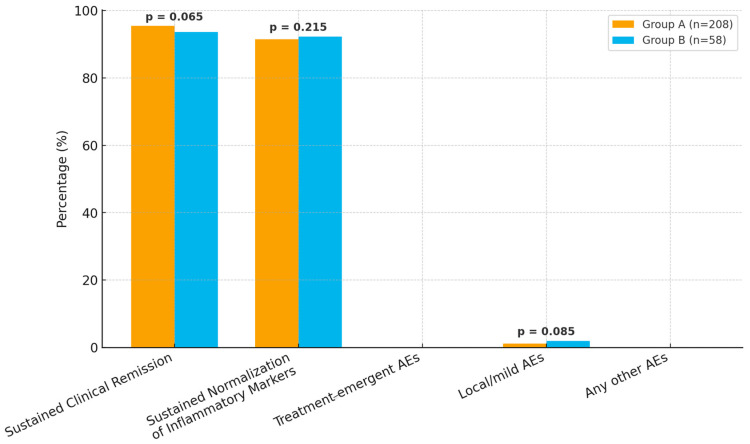
Primary and secondary outcomes in groups A and B.

Sustained clinical remission was achieved in 198 (95.4%) of group A and 54 (93.6%) of group B participants (Figure 2). Sustained normalization of inflammatory markers was also comparable, occurring in 190 (91.5%) of group A and 54 (92.3%) of group B participants. Inflammatory biomarker levels were generally comparable between CD and UC. C-reactive protein (CRP) was slightly higher in CD than UC (8.3 vs. 7.8 mg/L). In contrast, fecal calprotectin levels were higher in UC compared with CD (142 vs. 118 µg/g). Serum albumin concentrations were similar between groups (40 g/L in CD vs. 41 g/L in UC). All values indicate sustained normalization of inflammatory markers.

### 3.3. Predictors of Biosimilar Switch

**Figure 3:**
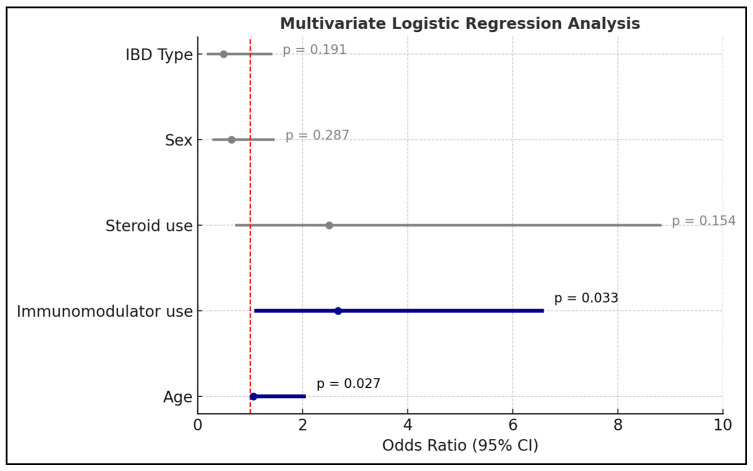
Factors associated with biosimilar switching.

The regression model showed a significant positive association between age and biosimilar switching (Figure 3). Each additional year of age was associated with a 3% increase in the odds of switching (OR = 1.03; CI = 1.05–2.06; *p* = 0.027). Additionally, the use of immunomodulators prior to starting the biosimilar was also a significant predictor. Patients who had used immunomodulators prior to initiating biosimilar therapy had higher odds of switching the biosimilar therapy compared to those who had not (OR = 2.67; CI = 1.08–6.59; *p* = 0.033). Other variables in the cohort analysis, sex, disease type, and steroid use within the preceding 12 months of starting the biosimilar therapy, were not significantly associated with switching.

## 4. Discussion

This study investigated the efficacy and safety of single and multiple switching from biologic originators to biosimilars in patients with IBD. Our results demonstrated comparable rates of sustained clinical remission and normalization of inflammatory markers, indicating strong and consistent therapeutic efficacy in patients who were non-medically switched from adalimumab originator (Humira^®^) to adalimumab biosimilar and in those who non-medically switched back to the adalimumab originator.

Adverse events were minimal, with no serious or treatment-related complications reported, reflecting excellent tolerability across both groups. In the regression analysis, older age and prior use of immunomodulators were identified as significant predictors of biosimilar switching, while sex, disease type, and prior steroid use showed no significant association.

The demographic profile of our cohort is consistent with established epidemiological patterns of IBD, particularly the earlier onset typically observed in patients with CD [14]. The higher proportion of patients with CD compared to patients with UC in our study reflects the complex therapeutic landscape often encountered in CD management, where biologic therapy utilization tends to be more frequent due to the nature of the disease and higher rates of complications requiring advanced therapeutic interventions. Patients with CD typically require earlier initiation of biologic therapy due to the progressive, penetrating nature of the disease, whereas patients with UC may initially respond to conventional therapies before requiring biologic intervention.

The safety of biosimilar switching was assessed in a previous study [15]. The findings underscored the critical importance of enhanced patient monitoring during switching periods, individualized risk assessment, and the implementation of comprehensive safety management protocols in IBD to identify and promptly address adverse events that may compromise treatment continuation. The study reported that adalimumab-atto showed favorable tolerability. The absence of serious adverse events directly attributable to the biosimilar switch aligns with our findings. The NOR-SWITCH study, which included more than 1500 patients with multiple immune-mediated inflammatory diseases, including IBD, reported a lack of worsening of disease or treatment discontinuation after a non-medical switch from originator adalimumab to biosimilars [16]. Similarly, a large Danish cohort study conducted in patients with psoriasis demonstrated that switching to adalimumab biosimilars maintained treatment effectiveness without an increase in adverse events [17]. Together, these data suggest that non-medical switching to biosimilars is safe and effective across a range of immune-mediated conditions, including IBD.

Furthermore, a comprehensive real-world study conducted across five major European countries involving 375 patients with CD and UC demonstrated that adalimumab-atto delivers comparable clinical outcomes to its reference product, with particularly impressive results showing 74% of treatment-naive patients and 89% of patients who switched from Humira^®^ achieving clinical remission [18]. Notably, the primary driver for switching from Humira^®^ to adalimumab-atto was cost-effectiveness rather than clinical concerns, and the transition proved safe and effective with no significant differences in outcomes between patients who initiated adalimumab-atto versus those who switched from the reference product, providing reassurance to both healthcare providers and patients about the biosimilar’s clinical performance in routine practice. These real-world findings are supported by the PERFUSE study, which examined multiple switches between the originator infliximab and the biosimilar CT-P13 in patients with IBD. The study demonstrated that repeated transitions did not result in loss of clinical response, increased adverse events, or emergent safety issues. The PERFUSE study concluded that multi-switching between the biosimilar and reference product is safe [19].

The favorable safety profile observed with adalimumab biosimilars (adalimumab-adaz and adalimumab-atto) may be partly attributed to the molecular structure of adalimumab, which is a fully human monoclonal antibody and therefore inherently less immunogenic. In contrast, infliximab and its biosimilar CT-P13 are chimeric antibodies containing approximately 25% murine sequences. This chimeric composition introduces a higher theoretical potential for immunogenicity, particularly during treatment transitions. Although clinical trials and real-world studies consistently demonstrated comparable immunogenicity between infliximab originators and their biosimilars, the structural differences may still contribute to increased susceptibility to immune recognition during multiple-switching scenarios [20].

The findings of our study provide evidence supporting the safety and efficacy of adalimumab biosimilars in patients with IBD who were in remission on the originator product. This supports non-medical switching practices as a feasible strategy in routine clinical care, potentially reducing treatment costs without compromising patient outcomes. The comparable rates of sustained clinical remission and normalization of inflammatory markers suggest that biosimilars can maintain disease control. Additionally, the low incidence of treatment-emergent adverse events reinforces their favorable safety profile. Overall, these results can guide clinicians, pharmacists, and policymakers in optimizing biologic use and resource allocation in IBD management. Our study findings are relevant in the growing landscape of biosimilar use in IBD. A biosimilar is defined by the U.S. Food and Drug Administration (FDA) as a biological product that is highly similar to an already-approved reference product, with no clinically meaningful differences in safety, purity, and potency [21]. In the European Union, biosimilars undergo rigorous evaluation by the European Medicines Agency (EMA), requiring comprehensive comparability studies demonstrating similarity in physicochemical characteristics, biological activity, efficacy, safety, and immunogenicity [22]. Similarly, the FDA requires extensive analytical, preclinical, and clinical data to establish biosimilarity. Infliximab biosimilars (CT-P13 and SB2) were among the first to gain regulatory approval and clinical adoption, followed by adalimumab biosimilars (adalimumab-atto and adalimumab-adaz, among others). More recently, biosimilars for vedolizumab and ustekinumab have entered development pipelines, promising further therapeutic options. The introduction of these biosimilars has resulted in cost reductions of 30–70% compared to originator products, significantly improving access to biologic therapy, particularly in resource-limited healthcare systems.

No evident negative impact from biosimilar interchangeability on clinical safety outcomes was observed in patients with IBD who underwent a switch from the originator drug to a biosimilar drug, as explained by D’Amico et al. [23]. Similarly, a study comparing adalimumab-atto and adalimumab-adaz found no significant increase in hospitalization or surgery [24]. Our data showed no rise in serious adverse events following multiple switches. Similarly to our finding, Gros et al. found that multiple successive switches from infliximab originator to CT-P13 are effective and safe in patients with IBD, irrespective of the number of infliximab switches [25]. This supports the overall safety of interchangeability among adalimumab biosimilars in patients with CD.

The present study has several clinical implications. The comparable rates of sustained clinical remission and normalization of inflammatory markers between the groups suggest that switching does not compromise disease control, providing reassurance to clinicians, pharmacists, and patients. The minimal incidence of adverse events and absence of serious safety concerns further emphasize the tolerability of biosimilars in routine practice. Additionally, the identification of older age and prior immunomodulator use as predictors of switching indicates that patient selection and monitoring strategies can be refined to ensure safe implementation. Considering the significant cost savings associated with biosimilars, these findings support their integration into national formularies and treatment pathways as part of value-based healthcare delivery in inflammatory bowel disease.

This study has several notable strengths. It was a multicenter study conducted in specialized tertiary gastroenterology centers, enhancing the generalizability of the findings. The study adhered to the STROBE reporting guidelines, ensuring methodological rigor and transparency. The inclusion and exclusion criteria were clearly defined, and disease classification and remission criteria were based on validated international tools, ensuring diagnostic consistency. Comprehensive baseline and clinical data collection allowed for detailed subgroup analyses and adjustment for potential confounders. The study also examined clinically relevant groups reflecting real-world non-medical switching practices, with both safety and efficacy outcomes objectively defined. In addition, the use of robust statistical analyses (chi-square and logistic regression) further strengthened the validity and reliability of the results. Finally, by evaluating the clinical effectiveness and safety of biosimilar switching in a Middle Eastern IBD population, this study contributes important region-specific evidence that supports the broader generalizability of international findings and reinforces the value of biosimilars in routine clinical practice within this geographic setting.

Despite these strengths, this study has some limitations. The observational nature of the study and lack of randomization may introduce bias and potential confounders. Adverse event reporting relied on clinical observation and patient-reported symptoms, which could result in underreporting. Furthermore, the predominance of CD patients (88.6%) in the cohort may limit the generalizability of the findings to UC populations. However, adalimumab is more commonly used in routine clinical practice for the management of CD than UC, which likely explains the lower number of patients with UC compared to those with CD in our study.

## 5. Conclusions

Multiple switches of adalimumab biosimilars can be safely undertaken without increasing the risk of adverse reactions or treatment failure. In our cohort, sustained clinical remission rates were comparable between single-switch (95.4%) and multiple-switch (93.6%) groups, with no adverse events reported in either group. Our findings align with growing international evidence supporting the safety and effectiveness of this strategy. Our study provides meaningful evidence to guide policy, resource allocation, and clinician confidence in biosimilar interchangeability as a sustainable therapeutic strategy in IBD management. Biosimilars represent a crucial tool for sustainable IBD care. As healthcare costs continue to rise, biosimilars offer significant cost savings while maintaining treatment quality and patient safety. The growing evidence supporting biosimilar safety and interchangeability is expected to accelerate their adoption, with future trends likely including broader non-medical switching policies and expanded biosimilar options as additional biologic patents expire. Long-term prospective studies with extended follow-up periods are needed to confirm the durability of treatment response and safety.

## Figures and Tables

**Table 1:** Baseline characteristics of the entire cohort.

Variables	Total, n = 237
Age (years), median (IQR)	32 (26–43.50)
Sex, n (%)	
Male	146 (61.6%)
Female	91 (38.4%)
IBD subtypes, n (%)	
Crohn’s Disease (CD)	209 (88.2%)
Ulcerative Colitis (UC)	28 (11.8%)
CD Subtypes	
L1: Ileal	108 (52%)
L2: Colonic	21 (10%)
L3: Ileocolonic	75 (36%)
L4: Upper gastrointestinal	4 (2%)
P: Perianal involvement	35 (16.8%)
B1: Inflammatory	98 (47%)
B2: Stricturing	44 (21%)
B3: Penetrating	67 (32%)
Perianal disease *	46 (69%)
UC Subtypes	
E1: Ulcerative proctitis	12 (21%)
E2: Left-sided colitis	21 (36%)
E3: Extensive colitis	26 (44%)
Time Since Diagnosis (years), median (IQR)	7.00 (3–12)
Previous Biologic Use, n (%)	
Yes	224 (94.5%)
No	13 (5.4%)
Duration of Biologic Medication Use (months), mean (SD)	65 (28–112)
Previous immunomodulator use, n (%)	99 (41.7%)
Previous corticosteroid use, n (%)	26 (11%)
Biosimilar switches, n (%)	
Adalimumab-adaz	204 (86.1%)
Adalimumab-atto	33 (3.9%)

* Percentage of perianal disease is calculated from the B3: penetrating disease subtype.

**Table 2:** Primary and secondary outcomes of study groups.

Parameter	Group A (n = 208)	Group B (n = 58)	*p*-Value
Sustained Clinical Remission	198 (95.4%)	54 (93.6%)	0.065
Sustained Normalization of Inflammatory Markers	190 (91.5%)	54 (92.3%)	0.215
Treatment-emergent AEs Infection Treatment discontinuation	0	0	-
Local/mild AEs	3 (1.2%)	1 (2%)	0.085
Any other AEs	0%	0%	-

## Data Availability

Data are available on request from the corresponding author due to local legal and ethical restrictions.

## Abbreviations

The following abbreviations are used in this manuscript:

CDAI	Crohn’s Disease Activity Index
CRP	C-reactive protein
IBD	Inflammatory bowel disease
CD	Crohn’s disease
UC	Ulcerative colitis
